# Gorlin-Goltz Syndrome: A Case Series

**DOI:** 10.7759/cureus.45656

**Published:** 2023-09-21

**Authors:** Ameera Salahudheen, Naqoosh Haidry, Ejaz A Mokhtar, Peeyush Shivhare, Vyakhya Gupta

**Affiliations:** 1 Oral and Maxillofacial Surgery, All India Institute of Medical Sciences, Patna, Patna, IND; 2 Dentistry, All India Institute of Medical Sciences, Patna, Patna, IND; 3 Dentistry/Oral and Maxillofacial Surgery, All India Institute of Medical Sciences, Patna, Patna, IND

**Keywords:** marsupialization, indian, palmer planter pits, odontogenic keratocyst, gorlin-goltz syndrome

## Abstract

Gorlin-Goltz syndrome (GGS) among Indians is rarely reported. Since 1960, only 38 cases having 48 patients of Gorlin-Goltz syndrome have been identified in the Indian population. It is crucial to diagnose this illness early because it can be connected to a malignant lesion like fibrosarcoma, leiomyosarcoma or rhabdomyosarcoma. The four patients in this case series were identified and treated in our department between 2019 and 2023. The average patient age was around 20 years old. Jaw swelling and tooth movement were the two most typical presenting concerns. Odontogenic keratocysts (100%), palmer pits (100%), plantar pits (50%), calcification of falx cerebri (50%), and rib abnormalities (50%), were the most prevalent characteristics. None of the patients had basal cell cancer, cleft lip, or medulloblastoma.

Multiple odontogenic keratocysts were present in three cases, whereas a single odontogenic keratocyst (OKC) was seen in one patient. Patients were managed with either marsupialization or enucleation, depending on the size of the cyst. Two cases with a large cyst size were marsupialized by using a modified obturator. Two cases with small cysts were managed with enucleation of the cyst followed by chemical cauterization. Recurrence was seen in two cases. In one patient, we noticed the formation of a new cyst.

A GGS diagnosis can be made by having a systemic evaluation of the patient. A thorough examination of the patient should be performed in every histopathology-diagnosed case of OKC. This will help to miss the syndromic cases. The treatment part should be conservative, like marsupialization with an obturator in a large cyst. The obturator helps maintain patient hygiene and prevents regular visits for changing dressings. Small-sized cysts can be managed with enucleation and chemical cauterization. Radical resection should be avoided.

## Introduction

Gorlin-Goltz syndrome (GGS) is a multisystemic disorder that affects several tissues and organs of the diseased individual. Its prevalence varies with different ethnic groups. The syndrome consists of more than 100 diagnostic features [1]. These features were divided into major and minor diagnostic criteria by Evans et al., which were later modified by Kimonis et al. [2]. The presence of one major and three minor criteria, or two major and one minor criteria, is necessary to establish the diagnosis.

The characteristic features of the syndrome vary globally because of difference in penetrance and expressivity. Odontogenic keratocyst (OKC), palmer plantar pits, bifid ribs, and calcification of flax cerebri are the most common features in the Indian population [3]. Medulloblastoma is not reported in the Indian population as this syndrome presents difference in penetrance and expressivity. However, medulloblastoma is usually associated with the syndrome in other parts of the world. The characteristic features arise in the first, second, third, and fourth decades of life. It is important to diagnose the syndrome in early life, as the affected individuals are suspectable of aggressive basal cell carcinoma [4]. Multiple OKCs, palmer planter pits, frontal bossing, cleft lip, and palate are usually seen in the first and second decades of life. The clinician should be aware of the signs and symptoms that are present in the first and second decades of life. Usually, multiple OKCs are seen in patients associated with this syndrome. However, a solitary OKC has also been reported in the literature [5].

Very few cases of GGS had been reported previously in the Indian literature, probably representing under-recognition. The purpose of this case review is to describe in detail the clinical, radiological, and mode of management of four cases managed in our department.

## Case presentation

Here we report a case series of four patients who were diagnosed and managed in our department from 2019 to 2023.

Case 1

**Figure 1:**
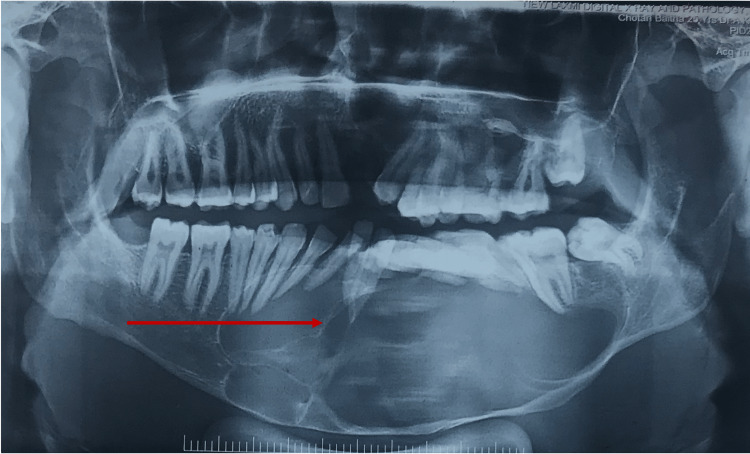
Orthopantomogram showing a large multilocular radiolucent lesion in the mandible with pathological migration of teeth, also external root resorption of anterior mandibular incisor

A 25-year-old male patient reported to the outpatient department at the All India Institute of Medical Sciences (AIIMS) Patna with swelling in the lower jaw for eight months. There was no previous history of surgery. An orthopantomogram (OPG) showed a single large multilocular radiolucency in the mandible from tooth number 45 to 35 (Figure 1).

**Figure 2:**
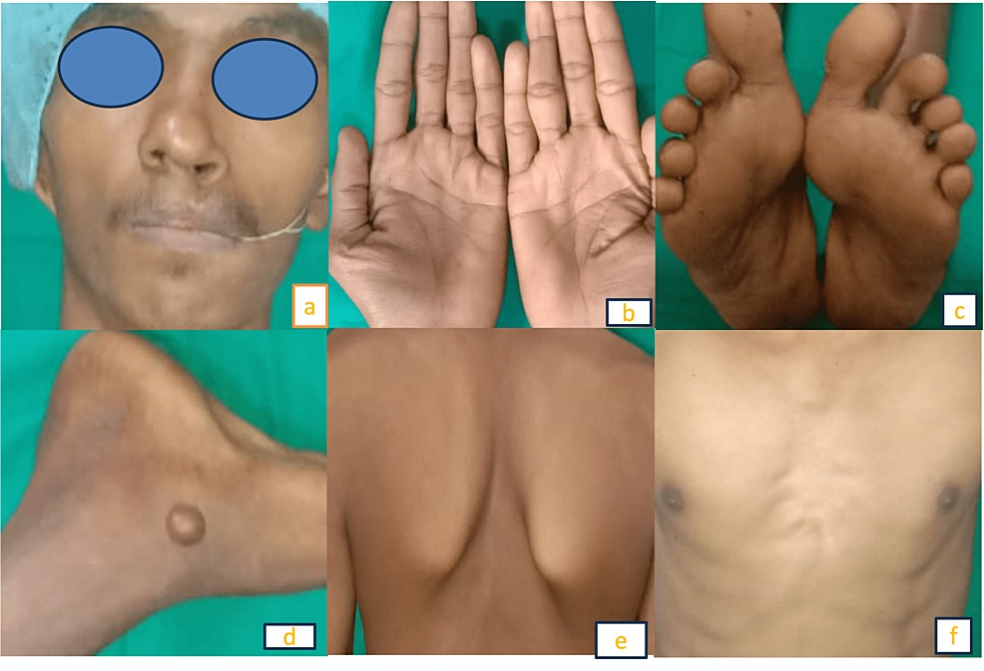
Clinical features seen in patient. (a) cleft lip, frontal bossing, increased intercanthal distance; (b, c) palmer planter pits; (d) epidermoid cyst; (e) Sprengel’s deformity; (f) pectus deformity

Needle aspiration from the cystic cavity showed cheesy discharge. On general examination, palmers (>20 pits) and planters (>5 pits) were seen. The patient also had macrocephaly, frontal bossing, increased intercanthal distance (46 mm), epidermoid cyst, pectus deformity and an elevated scapula (Figure 2a-2f).

**Figure 3:**
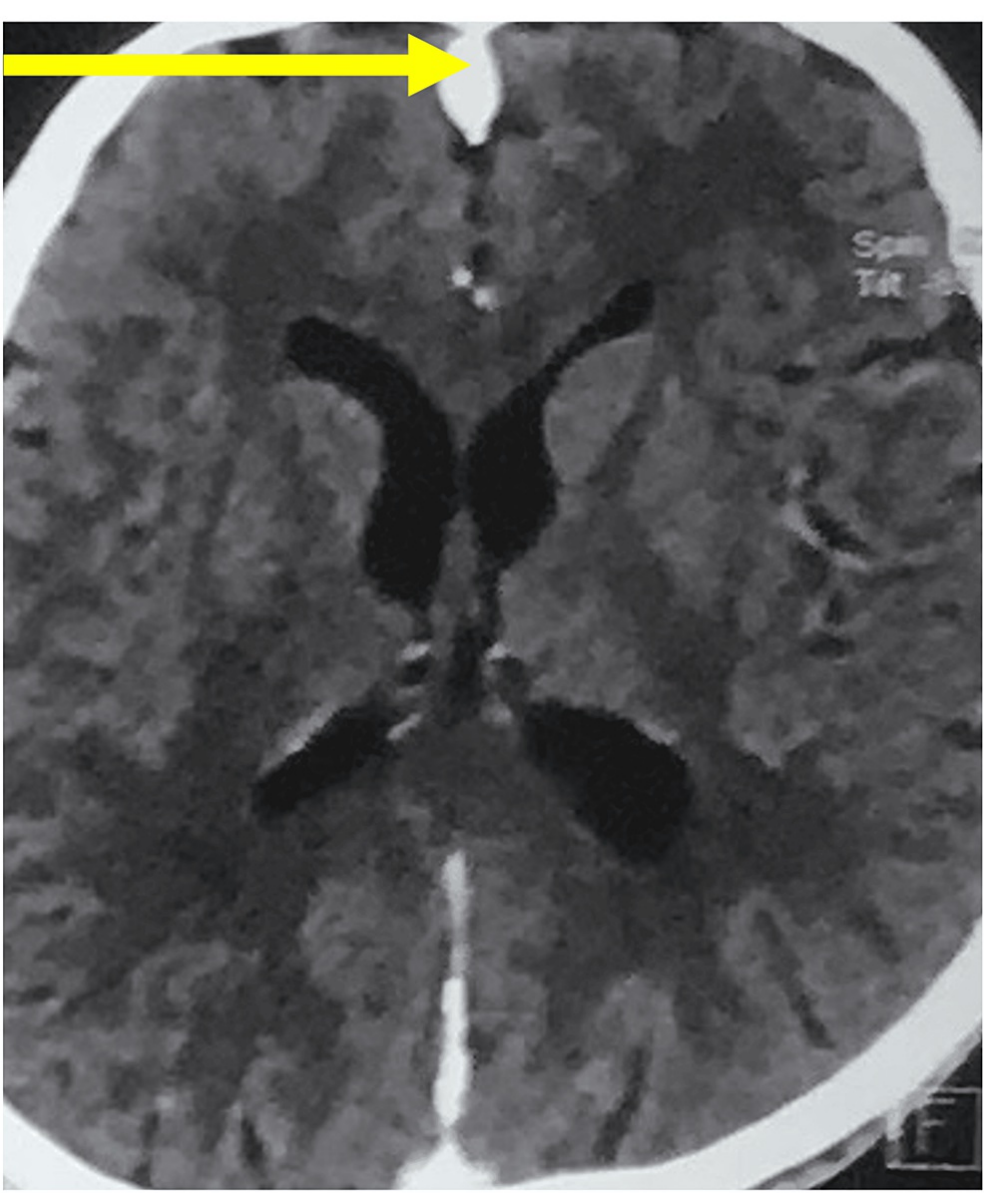
CT Axial section brain showing calcification of falx cerebri

The computed tomography (CT) head showed calcification of the falx cerebri (Figure 3).

**Figure 4:**
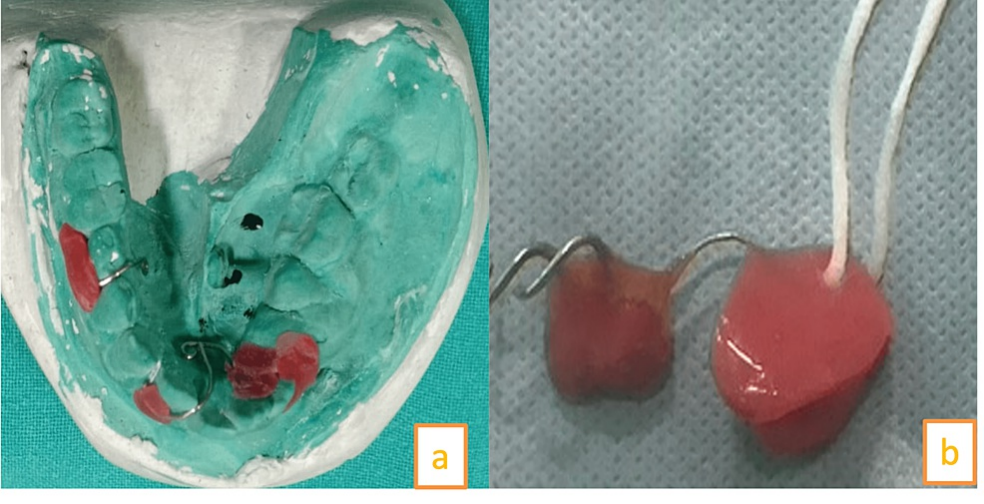
(a) Model of the cystic cavity with wire adaptation to form flexible obturator so that it can go in the cystic cavity, (b) modified obturator with two acrylic parts: one part can go in the cystic cavity while the other acrylic part to provide stability

Bifid ribs were seen in chest X-ray. An ultrasound was performed to rule out cardiac fibroma. Based on clinical criteria, a diagnosis of Gorlin-Goltz was made. As the cyst was large, marsupialization was performed. The cystic lining was sent for histopathological examination. Histopathologic examination confirmed OKC. Two weeks after marsupialization, an impression of the cystic cavity was made. A customized obturator was made. The obturator was made with 20-gauge wire and cold-cure acrylic (Figure 4a, 4b).

**Figure 5:**
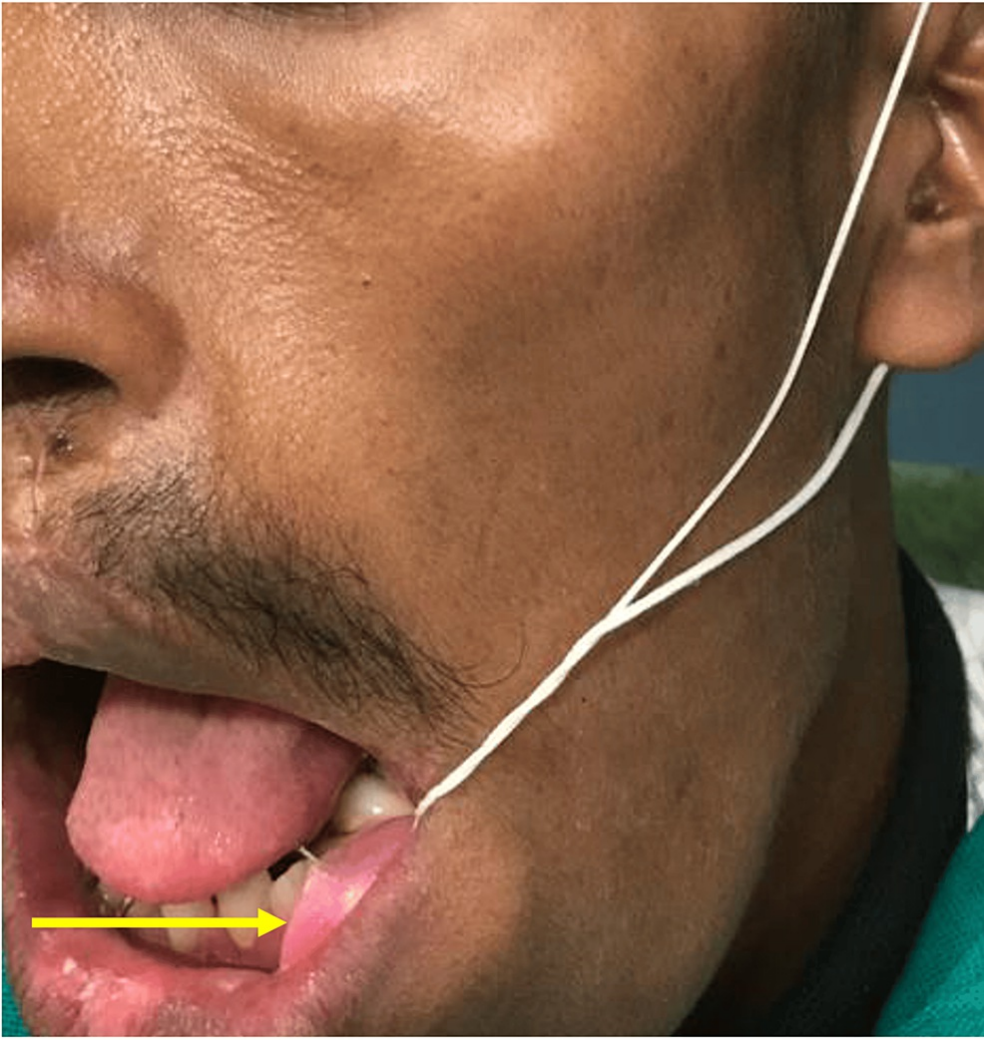
Obturator placed in the marsupialized cavity with cord attached

The obturator was compact and it was connected with a thick thread and the thread could be attached to pinna at the time of sleeping (Figure 5).

**Figure 6:**
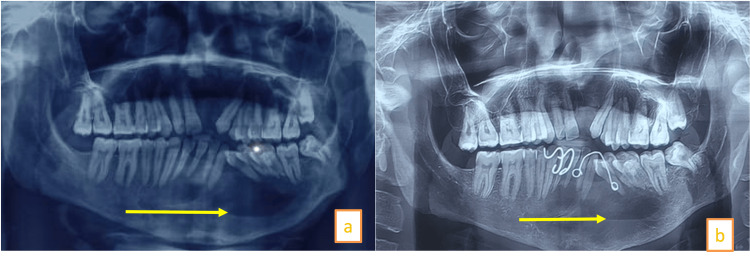
(a) OPG at six-month follow-up showing a reduction in the size of the cystic cavity. (b) 1.5-year follow-up OPG showing a significantly reduced cystic cavity OPG: Orthopantomogram

This prevents the obturator from being swallowed by the patient. Regular irrigation of the cystic cavity with betadine and normal saline was advised. After six months of follow-up, the size of the cystic cavity was reduced. A 1.5-year follow-up showed a significantly reduced cystic cavity (Figure 6).

**Figure 7:**
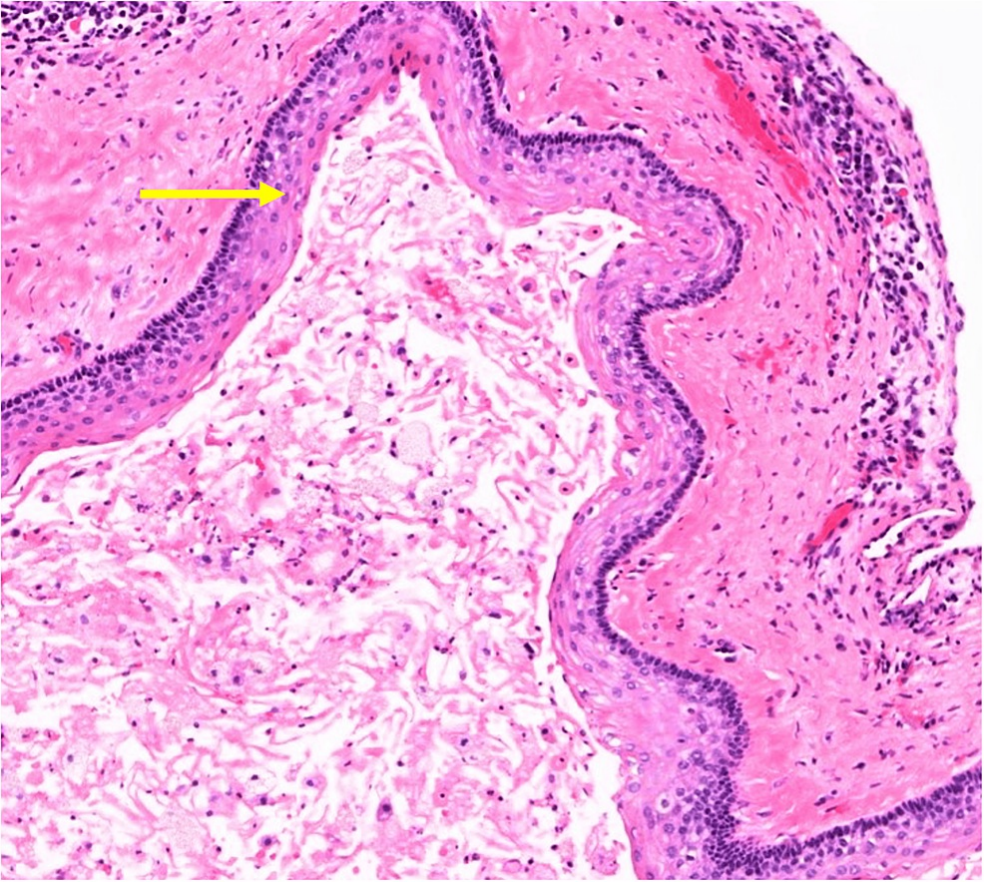
Cystic cavity lined by parakeratinised stratified epithelium of 6-8 cell thickness

The patient is being planned for enucleation of the marsupialized cyst. A new cyst formation was seen on the left side of the maxilla at the one-year follow-up. This cyst was managed with enucleation. Histopathology examination confirmed odontogenic keratocyst (Figure 7).

Case 2

**Figure 8:**
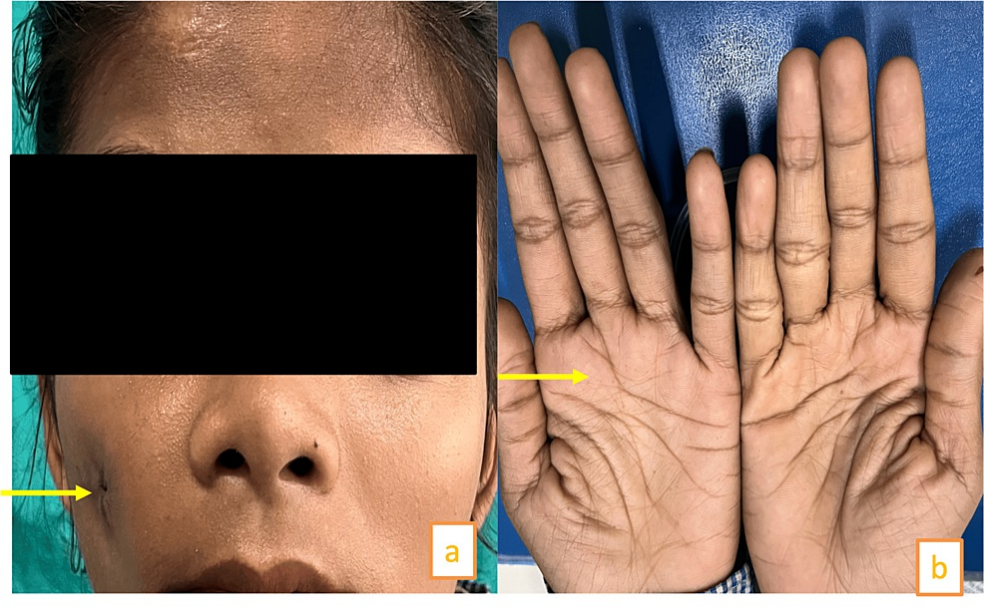
(a) Extra oral sinus. (b) Palmer pits

A 17-year-old female reported swelling in the right side of her upper jaw for four months. She had previously undergone surgery 1.5 years ago. On clinical examination, the patient had extra oral sinuses (Figure 8a).

**Figure 9:**
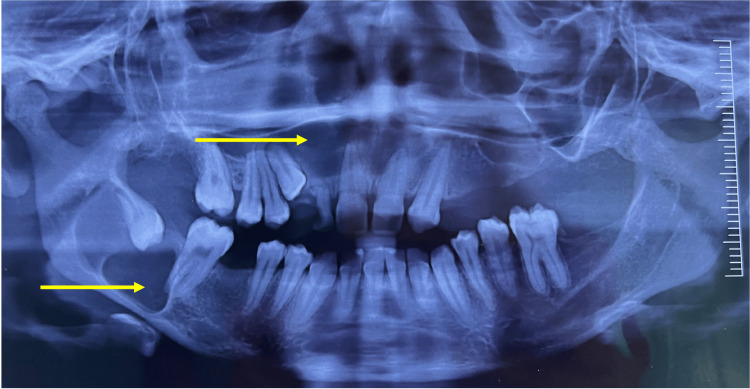
Orthopantomogram showing multiple cysts involving the mandibular body and ramus on the right side, right-side maxilla and in the maxillary midline

OPG showed multiple cysts involving the mandibular body and ramus on the right side and on the right side of the maxilla (Figure 9).

On general examination, the patient also had palmer planter pits (>10 pits), frontal bossing, and an elevated scapula (Figure 8b). An incisional biopsy followed by histopathological examination confirmed OKC. Based on major and minor criteria, Gorlin-Goltz syndrome was confirmed. The patient was managed with enucleation of the cystic lesion and chemical cauterization. Two years of follow-up showed no recurrence.

Case 3

**Figure 10:**
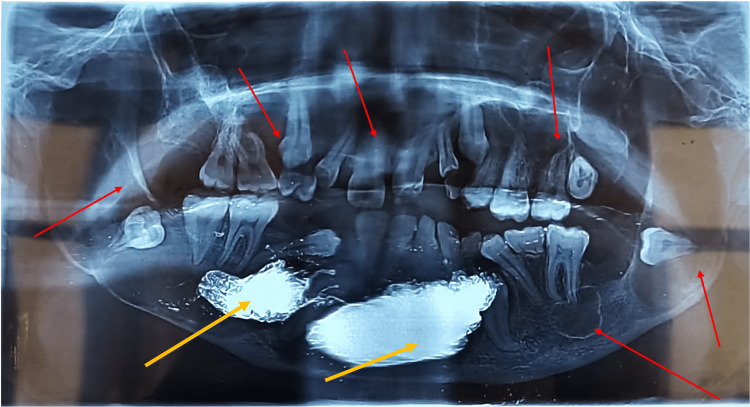
Yellow arrows in orthopantomogram showing two sites of marsupialized area and red arrows showing multiple cysts both in the maxilla and mandible

A 20-year-old male reported swelling of the bilateral mandible with iodoform gauze in place. He had gone through marsupialization at a private clinic three months ago. OPG showed two sites of marsupialized areas and multiple cysts both in the maxilla and mandible (Figure 10). On careful radiographic examination, three additional cysts were seen in the mandible which were not addressed.

**Figure 11:**
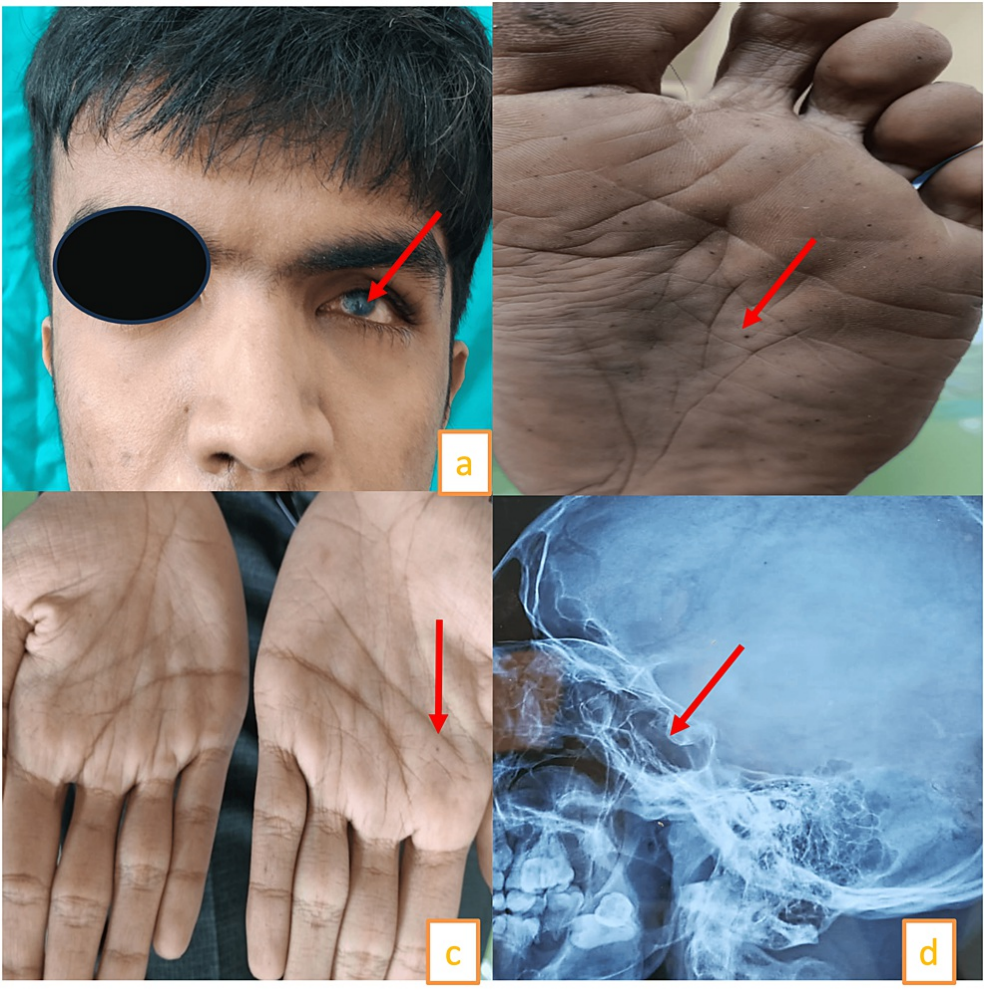
(a) Pterygium of conjunctiva in the left eyeball. (b) Palmer pits. (c) Plantar pits. (d) Arrow showing birding of sella turcica.

In addition, the patient had three cysts in the maxilla. On general examination, the patient was congenitally blind on the left side. In addition, the patient had palmer plantar pits, frontal bossing, increased interpupillary distance, and macrocephaly. Radiographic analysis showed the patient had a bifid rib, scoliosis, and falx cerebri calcification (Figure 11a-11d).

Histopathological examination confirmed OKC. Based on features, Gorlin-Goltz syndrome was confirmed. The maxillary cyst was managed with enucleation, while marsupialization was done for the mandibular cyst, and the patient is currently under treatment.

Case 4

**Figure 12:**
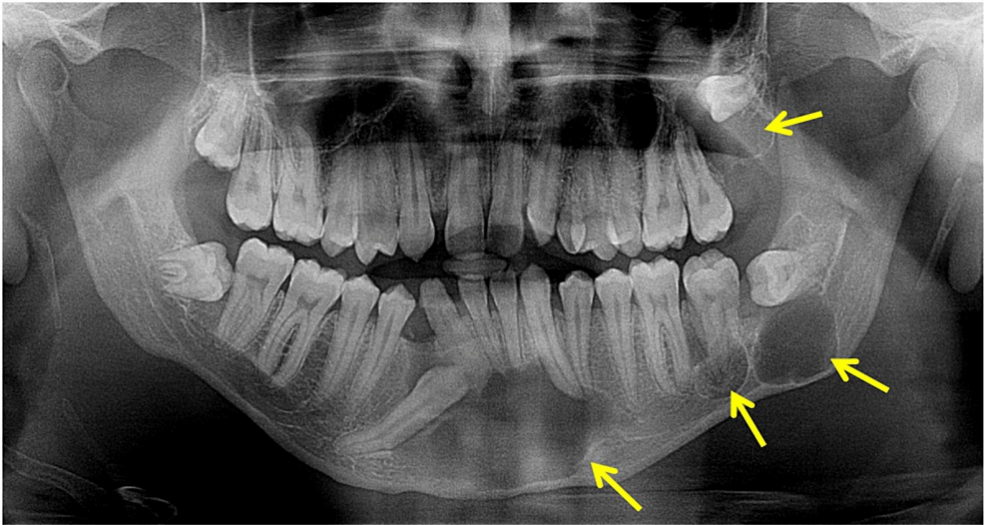
Orthopantomogram showing multiple cysts both in the mandible and one cyst in the maxilla

A 20-year-old male reported swelling for six months on the left side of the mandible. OPG showed three cystic lesions in the mandible and one cyst in the maxilla (Figure 12).

**Figure 13:**
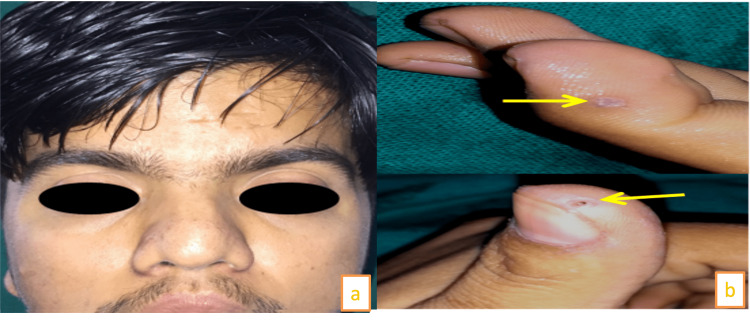
(a) Clinical features in the patient: frontal bossing, increased intercanthal distance. (b) Arrows showing finger pits

The clinical features included finger pits and frontal bossing (Figure 13a, 13b).

The radiological findings included calcification of falx cerebri and bifid ribs. Histopathological examination revealed OKC. Based on major and minor criteria, Gorlin-Goltz syndrome was confirmed. The patient was managed with enucleation of the cyst and chemical cauterization. After a year follow-up recurrence was seen in the left side of the mandible which was managed with enucleation. The patient was managed with enucleation of the cyst and chemical cauterization.

The clinical and radiological findings of all the cases are summarized in Table 1.

**Table 1:** Table including clinical, radiographic features, mode of management and period of follow-up of patient. IPD: Interpupillary distance

Parameters	Case 1	Case 2	Case 3	Case 4
Age (years)	25/M	17/F	20/M	20/M
Site involved	Cyst involving mandibular body bilateral	Cyst involving mandibular body + ramus right side + right side maxilla and maxillary midline	Cyst involving mandibular ramus bilateral + mandibular body bilateral + bilateral maxilla	Cyst involving mandibular ramus bilateral + left maxilla
Total number of cysts present	2	3	8	4
Other clinical findings	Palmer planter pits (>20 pits), sebaceous cyst, frontal bossing, pectus deformity, elevated scapula	Palmer pits (>10 pits), frontal bossing, elevated scapula	Palmer planter pits (>10 pits), frontal bossing, congenital blindness of left eye (since birth), increased IPD, macrocephaly	Finger pits, frontal bossing
Radiographic findings	Cyst in bilateral mandible, Bifid ribs, falx cerebri calcification	Cyst in mandible (1) and maxilla (2), falx cerebri calcification	Cyst in mandible (4), falx cerebri calcification, bifid ribs, scoliosis	Cyst in bilateral mandible + maxilla, Bifid ribs, falx cerebri calcification
Mode of management	Marsupialisation followed by enucleation + chemical cauterisation	enucleation + chemical cauterisation	Marsupialisation followed by enucleation + chemical cauterisation	enucleation + chemical cauterisation
Follow up	3 years	2 years	2.5 years	2 years
Formation of new cyst	1 (maxilla), managed with enucleation + chemical cauterisation	No	1	2

## Discussion

Gorlin-Goltz syndrome (GGS) is an autosomal dominant condition. It is usually associated with a mutation in patched 1 gene located on chromosome 9. The prevalence of the syndrome is between 1:57,000 and 1:256,000 [6]. Evans et al. reported an incidence of 1 per 560,000 in the United Kingdom, while the estimated incidence in Australia and Italy is 1 per 64,000 and 256,000, respectively [7]. In India, only nine case series have been reported so far [3]. The male-to-female ratio is 1:1. We report four cases of Gorlin-Goltz syndrome. In our study, the male-to-female ratio was 3:2.

This syndrome was first described in the literature by Gorlin and Goltz in 1960. It has a triad of odontogenic keratocysts, basal cell carcinoma, and bifid ribs [8, 9]. Rayner et al. added additional criteria, including calcification of the falx cerebri and palmer/plantar pits [10]. Kimonis and Evans divided the features into major and minor criteria [7]. The major criteria are histologically proven OKCs of the jaws, palmar or plantar pits (three or more), multiple basal cell carcinomas or one occurring under the age of 20 years, bilamellar calcification of the falx cerebri, bifid, fused, or markedly splayed ribs, and first-degree relatives with naevoid basal cell carcinoma syndrome. The minor criteria are more than 100 (Table 2) [11-15].

**Table 2:** Table showing numerous Gorlin-Goltz syndrome's features included in minor criteria

1	Congenital malformation	Cleft lip and palate, congenital blindness, congenital cataract, syndactyly, postaxial polydactyly
2	Craniofacial features	Frontal and biparietal bossing, hypertelorism, macrocephaly determined after adjustment of height
3	Abnormalities associated with skin	Coarse faces, facial milia, sebaceous cyst, epidermal cyst
4	Other skeletal deformity	Skeletal deformity: Sprengel deformity, extra ribs, pectus deformity
5	Abnormalities associated with eye	Coloboma of iris, choroid and optic nerve, glaucoma, microphthalmia, pigmentary changes of retinal epithelium, strabismus and nystagmus, orbital cyst
6	Radiographic abnormalities	Bridging of sella turcica, vertebral anomalies such as hemivertebrae, fusion or elongation of the vertebral bodies, modeling defects of hand and feet or flame-shaped hand and feet, kyphoscoliosis, short fourth metacarpal, mandibular coronoid process are enlarged.
7	Neurological features	Medulloblastoma and brain tumor and seizure: astrocytoma, meningioma, craniopharyngioma, oligodendroglioma, cysts of the brain have been reported: colloid cyst of the third ventricle, arachnoid cyst, intraparenchymal cyst, cysts of the septum pellucidum.
8	Gynaecological features	Ovarian fibroma and fibrosarcoma
9	Gastrointestinal features	Lymphomesentric cyst
10	Urological features	Kidney anomalies: These have included horseshoe kidney, L-shaped kidney, unilateral renal agenesis, renal cyst, and duplication of renal pelvis and ureters
11	Cardiac features	Cardiac fibroma
12	Genital features	Hypogonadism
13	Pulmonary features	Pleural cyst
14	Miscellaneous other tumours	There appears to be an increased incidence of several other neoplasms or hamartomas: leiomyomas of bowel and mesentery, leiomyosarcoma, adrenal cystic lymphangioma, thyroid adenoma, lymphangiomyoma, melanoma, ameloblastoma, craniopharyngioma, mesenchymoma, Hodgkin's lymphoma, non-Hodgkin's lymphoma, rhabdomyosarcoma, nasal dermoid, seminoma, paratesticular pseudotumor, schwannoma, pleomorphic adenoma of parotid, adenoid cystic carcinoma of the salivary gland, and adrenal tumors. Fetal rhabdomyomas are also reported.

The presence of one major and three minor criteria, or two major and one minor criteria, is necessary to establish the diagnosis of Gorlin-Goltz.

The various characteristic features seen in the Indian population are OKC, palmer plantar pits, bifid ribs, and calcification, which are the most common features. Medulloblastoma is not reported in the Indian population [3]. The percentage of various features in the Indian population is bifid ribs (30-60%), calcification of falx cerebri (37-79%), calcification of falx cerebri, multiple OKC (95.8%), rib anomalies (71%), frontal bossing (45.8%), basal cell carcinoma (41%), macrocephaly (25%), and polydactyly/syndactyly (21%). Among the various clinical findings, OKC is usually the first one to get diagnosed, as it is usually the first sign and symptom patients experience when they first visit the dentist. Moreover, the incidence of OKC in GGS patients is around 80%.

In our case, OKC was seen in 100% of the cases. Multiple OKCs were seen in 75% of cases, whereas a single OKC was seen in 25% of cases. In 25% of the cases, a new OKC formation was seen. Palmer pits and frontal bossing were seen in 100% of cases. Plantar pits were seen in 50% of cases. The sebaceous cyst was seen in 25% of cases. Bifid rib was seen in 50% of cases, and falx cerebri calcification was seen in 50% of the cases. In one of our cases, congenital blindness was seen. Congenital blindness is rarely reported in GGS. It was seen in one of our cases. Basal cell carcinoma is mostly reported in young patients; ovarian fibroma is reported in patients mostly at the age of 16-45 years; and medulloblastoma is usually reported within the first two years of life.

The recurrence rate of OKC associated with the syndrome is around 82%, compared to 61% in non-syndromic patients [16]. Donatsky and Hjørting-Hansen [17] reported a significantly higher presence of proliferative epithelial remnants in the connective tissue wall of the cyst, which might be the cause for a higher recurrence. We have also noticed recurrences in two of our cases.

The management of Gorlin-Goltz syndrome requires a multidisciplinary approach. It may require a maxillofacial unit, a pediatric unit, a cardiac unit, a gynecological unit, and a genetic counselor for the evaluation of different organs. The accepted treatment modalities for OKC associated with Gorlin-Goltz can be categorised into five types: 1. Decompression or marsupialization for OKC of large size, 2. Enucleation of OKC followed by mechanical curettage, 3. Enucleation followed by chemical cauterisation with Carnoy's solution, 4. Enucleation of cyst followed liquid nitrogen cryotherapy, 5. Block resection with or without preservation of jaw. Carnoy's solution is composed of 3 ml of chloroform, 6 ml of absolute ethanol, 1 ml of glacial acetic acid, and 1 g of ferric chloride. It is usually in conjunction with surgery to prevent recurrence rates after enucleation of cyst. It promotes chemical necrosis of up to 1.5 mm. It is useful in cases where the cyst is adjacent to neuro-vascular bundle or the lesion is abutting soft tissue. Moreover, as OKC is associated with satellite and daughter cyst, Carnoy's solution is effective in eliminating these small cysts. Although some paraesthesia is noted by the application of Carnoy's solution, however, it is temporary [18-20].

Small unilocular cysts are enucleated, whereas in larger cysts, marsupialization followed by enucleation is advised [7]. The percentage of postoperative recurrences with this method of treatment is significantly lower, as demonstrated by Chinese authors in a large group of patients [21]. According to our limited experience, we have noticed marsupialization helps reduce the size of lesions, thus preventing the morbidity associated with surgery if treated without marsupialization. Moreover, the use of a modified obturator, as used in our case, is an effective way to maintain patient hygiene and prevent regular visits.

## Conclusions

Gorlin-Goltz syndrome shows variability in penetrance and expressivity. It is important to be aware of the incidence and relative variability of certain diagnostic characteristics. This would help clinicians efficiently diagnose the syndrome. Patients with this syndrome can also develop new cysts or malignancies. Therefore, a regular visit is essential. The treatment of OKC associated with this syndrome should be conservative, like marsupialization or enucleation. Radical treatment should be avoided as it causes facial deformity.
